# Comparative analysis of novel esophageal pressure monitoring catheters versus commercially available alternatives in a biomechanical model of the thoracic cavity

**DOI:** 10.1038/s41598-024-59790-1

**Published:** 2024-04-29

**Authors:** Gabriella Abbate, Sebastiano Maria Colombo, Clayton Semenzin, Noriko Sato, Keibun Liu, Carmen Ainola, Angelo Milani, Gabriele Fior, Nchafatso Obonyo, Nicole White, Davide Chiumello, Jo Pauls, Jacky Y. Suen, John F. Fraser, Gianluigi Li Bassi

**Affiliations:** 1 Critical Care Research Group, The Prince Charles Hospital, Brisbane, Australia; 2 Department of Anesthesia Critical Care and Emergency, Fondazione IRCCS Ca’ Granda Ospedale Maggiore Policlinico, Milan, Italy; 3 School of Engineering and Built Environment, Griffith University, Southport, Australia; 4 Department of Biomedical Sciences, Humanitas University, Pieve Emanuele, Milan, Italy; 5 Institute for Molecular Bioscience, University of Queensland, Brisbane, Australia; 6 Initiative to Develop African Research Leaders/KEMRI-Wellcome Trust Research Programme, Kilifi, Kenya; 7 Australian Centre for Health Services Innovation and Centre for Healthcare Transformation, School of Public Health and Social Work, Queensland University of Technology, Brisbane, QLD Australia; 8 San Paolo Hospital, Milan, Italy; 9 School of Engineering and Built Environment, Griffith University, Brisbane, QLD Australia; 10 School of Pharmacy and Medical Sciences, Griffith University, Southport, Australia; 11 Intensive Care Unit, St Andrew’s War Memorial Hospital, Spring Hill, QLD Australia; 12 Intensive Care Unit, The Wesley Hospital, Auchenflower, QLD Australia; 13 Wesley Research Institute, Auchenflower, QLD Australia; 14 Critical Care Research Group, The Prince Charles Hospital, 627 Rode Rd, Chermside, QLD 4032 Australia

**Keywords:** Esophageal pressure, Nasogastric catheter, Trans-esophageal pressure, Mechanical ventilation, Intensive care unit, Preclinical research, Respiratory distress syndrome

## Abstract

Transpulmonary pressure can be estimated using esophageal balloon (EB) catheters, which come in a variety of manufacturing configurations. We assessed the performance of novel polyurethane EB designs, Aspisafe NG and NG+, against existing alternatives. We created a biomechanical model of the chest cavity using a plastic chamber and an ex-vivo porcine esophagus. The chamber was pressurized (− 20 and + 20 cmH_2_O) to simulate pleural pressures. We conducted tests with various EB inflation volumes and measured transesophageal pressure (TEP). TEP measurement was defined as accurate when the difference between pressure within the EB and chamber was 0 ± 1 cmH_2_O. We computed the minimal (V_accuracy-min_) and maximal (V_accuracy-max_) EB inflation volumes of accuracy. Inflation volumes were further validated using a surrogate method derived by the clinically validated positive pressure occlusion test (PPOT). When the esophageal balloons were filled with inflation volumes within the range provided by the manufacturers, the accuracy of TEP measurements was marginal. Our tests found median V_accuracy-min_ across EB of 0.00–0.50 mL (*p* = 0.130), whereas V_accuracy-max_ ranged 0.50–2.25 mL (*p* = 0.002). Post PPOT validation, median TEP was − 0.4 cmH_2_O (− 1.5 to 0.3) (*p* < 0.001 among catheters). The Aspisafe NG and NG+ were accurate in 81.7% and 77.8% of the measurements, respectively. We characterized two new EBs, which demonstrated good benchtop accuracy in TEP measurements. However, accuracy was notably influenced by the precise selection of EB inflation volumes.

## Introduction

Pleural pressure is the pressure surrounding the lungs and its measurement is essential to characterize the respiratory mechanical properties by partitioning the airway pressure into lung and chest wall pressures. In critical care and respiratory medicine, esophageal pressure is used as a surrogate for pleural pressure^1^. Indeed, it has been consistently demonstrated that dynamic variations in esophageal pressure reflect changes in pleural pressure^2^. The clinical implications of esophageal pressure are varied, including assessment of respiratory effort and work of breathing during spontaneous or assisted ventilation^3,4^, as well as assisting in the implementation of lung protective ventilation in patients with acute respiratory distress syndrome (ARDS)^5,6^.

Esophageal pressure is typically measured using an esophageal catheter with an air-filled balloon, placed in the mid-lower portion of the esophagus. The accuracy of this method relies on the correct transmission of the pressure surrounding the esophagus, namely the intrathoracic pressure, to the esophageal catheter balloon^7^. Several factors can affect the appropriate pressure transmission^1,8^, such as the elastic properties of the balloon and its appropriate air-filling, positioning of the balloon in the distal third of the esophagus, and the elastance of the esophagus^9–12^.

There are different types of esophageal balloon (EB) catheters available in the medical device market, each characterized by different length, diameter, compliance, and filling volume of the balloon. These characteristics influence the esophageal pressure measurement and must be considered to ensure an accurate estimate of pleural pressure^13,14^. The volume of air required for inflation varies across catheter types, and while underfilling leads to underestimation of esophageal pressure, overfilling can cause balloon stretching and pressure overestimation^10,15,16^. Moreover, it has been clearly demonstrated that the appropriate balloon filling volume varies under different external pressure conditions^12,13^.

Thus, a comprehensive understanding of the mechanical characteristics of EBs is crucial to appropriately use esophageal pressure monitoring catheters and to obtain valuable insights that can provide guidance for clinical practice. Several bench studies have previously analyzed commercially available EBs using different experimental lung models, revealing both inherent advantages and limitations^13–15,17^.

Recently, two catheters with integrated esophageal and gastric balloons have emerged in the medical market^17^. These catheters are primarily aimed at addressing gastro-esophageal reflux, by employing a sequential inflation approach of the esophageal and gastric balloons^18^. However, the EBs can also be used to measure esophageal pressure. Therefore, primary aim of this study was to study inflation volumes and appraise transesophageal pressure measurement accuracy of these new catheters, in comparison with commercially available alternatives. This evaluation was conducted employing an innovative biomechanical model of the thoracic cavity, purpose-built for this study. Secondary objectives encompassed the assessment of balloons dimensions, including length and size, alongside the characterization of their elastance properties.

## Materials and methods

### Ethics requirements

Prior to the initiation of this investigation, we engaged with the Queensland University of Technology Animal Ethics Committee. The Committee granted a waiver for approval, due to the utilization of esophagi sourced from a commercial slaughterhouse, where animals were not specifically euthanized for research objectives.

### Esophageal balloon catheters

Seven different types of commercially available EB catheters, characterized by balloons of varying dimensions and materials, were investigated (Fig. E1). In this study two novel catheters manufactured by Aspisafe were specifically examined: (1) Aspisafe NG+ and (2) Aspisafe NG (Aspisafe, NY, USA). The Aspisafe NG+ catheter features an advanced aspiration-feeding system and allows for sequential inflations of both the esophageal and gastric balloons to prevent gastro-esophageal reflux. These novel catheters were compared with five other catheters already available on the market: (3) SmartCath (CareFusion Co., San Diego, CA, USA), (4) SmartCath-G (CareFusion Co., San Diego, CA, USA), (5) Marquat (Marquat Genie Biomedical, Boissy-Saint-Léger Cedex, France), (6) Cooper (Cooper Surgical, Trumbull, CT, USA), and (7) Nutrivent (NutriVent Sidam, Modena, Italy). Prior to testing, each catheter was randomly selected for visual inspection, ensuring the exclusion of catheters with manufacturing defects. To verify air sealing, the balloon of each catheter was inflated underwater, and gentle pressure was manually applied to confirm the absence of air leaks. Of note, the Aspisafe catheters feature a single inflation channel for both the esophageal and gastric balloons. Therefore, before testing, the channel was clamped downstream of the EB to ensure proper sealing.

### Experimental setup

The study comprised various experimental phases to characterize accuracy in measuring external pressure, EBs dimensions and elastance (Table 1).

**Table 1:** Study assessments.

Esophageal balloon assessment	Esophageal catheter types (N)	Internal chamber pressure levels (N)	Esophageal balloon inflation volumes (N)	Test replication (N)	Total number of tests (N)	Analyzed values (N)
Outer diameter	7	N/A	1	3	21	21
Length	7	N/A	1	3	21	21
Elastance	7	N/A	Variable (Max internal balloon pressure of 40 cmH_2_O)	3	21	296
Appraisal of esophageal balloon volume for accurate transesophageal pressure measurement	7	9	Variable (Max internal balloon pressure of 30 cmH_2_O)	3	189	2032
Transesophageal pressure measurements accuracy post positive pressure occlusion testing	7	9	1	3	21	189

## Primary objective

### Appraisal of esophageal balloon filling volume for accurate transesophageal pressure measurement

**Figure 1:**
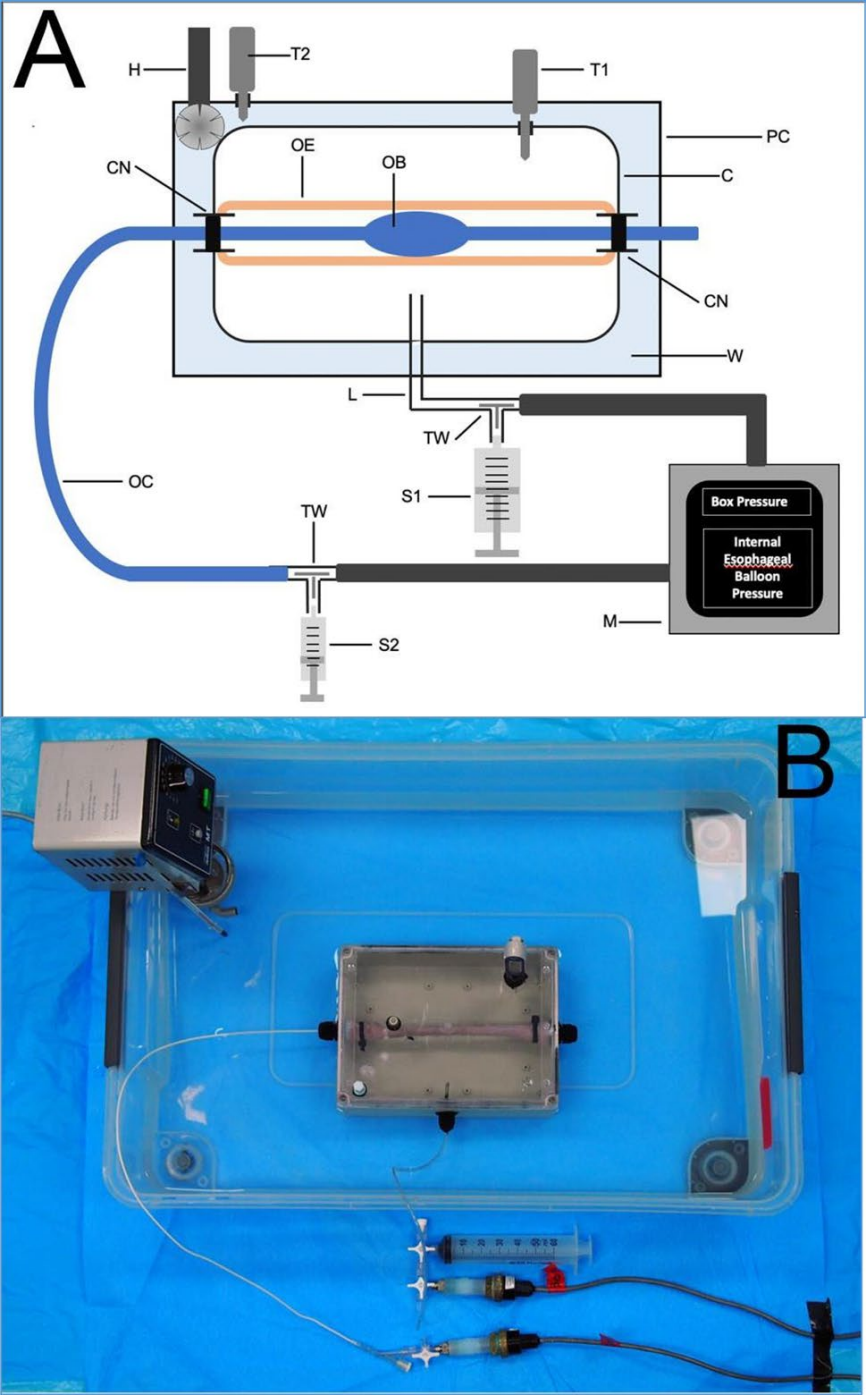
Figure depicts detailed sketch of ex-vivo experimental settings (**A**) to appraise transesophageal pressure and the setup during experiments (**B**). The ex-vivo model comprised a temperature- and pressure-controlled 2.5-L polycarbonate box (300Hx230Wx100D) (C) with a clear lid, which was filled with phosphate buffer solution through a PVC tube (L) via a 3-way stopcock (TW) connected with a 50-mL syringe (S1) and a pressure transducer, ultimately connected to a personal computer (M). The lateral ends of an esophagus of a 70–80 kg pig (OE) were mounted onto plastic reducers at the lateral ends of the box, and sealed with cable ties, as shown in (**B**). Finally, the esophageal catheter was inserted through the lateral washer nuts (CN) and the balloon (OB) advanced to the middle of the esophagus. This box was bathed into a temperature-controlled chamber (PC) filled with water (W), which was heated up to 38 °C by a water heater (H). Two temperature probes inside the box (T1) and inside the water chamber (T2) were used to monitor the box temperature and adjust the water heater. Finally, the esophageal catheter (OE) was connected to a 3-way stopcock (TW) and connected to a 3-mL syringe (S2) and a pressure transducer connected to the personal computer.

As shown in Fig. 1, a novel biomechanical model was developed to assess EB volumes and to measure transesophageal pressure (TEP). TEP measurement was defined as accurate when the difference between pressure within the EB and chamber fell within the range of − 1 to 1 cmH_2_O. The model comprised a pressure-controlled 2.5-L polycarbonate box (100 mm height × 300 mm width × 230 mm depth) with a clear lid, filled with phosphate buffer solution. An esophagus of 310 mm from a 70–80 kg pig was mounted in the box. The chamber was placed into a temperature-controlled bath to achieve a consistent temperature of 37 °C. The esophagi were harvested from pigs and preserved using a solution (Nasco-Guard Humectant Fluid, Nasco, Fort Atkinson, WI, USA) to maintain tissue elasticity, prevent tissue desiccation, and inhibit mold or bacterial growth. Two plastic reducer pieces were internally mounted at each site and connected to external cable glands, filled with insulation tubes. The lateral margins of the esophagus were mounted onto the plastic reducers and sealed with cable ties. Finally, the esophageal catheter was inserted through the cable glands, and the balloon was advanced to the middle of the esophagus. The pressure chamber comprised three additional openings: one to instill phosphate buffer solution and pressurize the internal environment, one to evacuate the chamber, and one to insert a digital stem thermometer. The esophageal catheter balloon was inserted and positioned in the middle of the esophagus, and its inflation line was connected via a 3-way stopcock to a 10 mL syringe for balloon inflation and to a pressure transducer. The box was also connected via a 3-way stopcock to a 50 mL syringe and a second pressure transducer. Pressure measurements were recorded using piezoresistive pressure transducers (PX181B–015C5V, Omega Engineering, Norwalk, CT, USA,—sensitivity 0.01 cmH_2_O, range + /− 1054.6 cm H_2_O) connected to a custom-made pressure box via 80-cm rigid tube lines. Signals were displayed continuously and recorded at a sample rate of 200 Hz through dedicated software. All parameters were visualized using ControlDesk software (dSPACE Ltd. Beech House, Melbourn Science Park, Melbourn, Australia). Pressure transducers were certified and calibrated following National Institute of Standards and Technology standards. During the experiments, the box pressure varied from − 20 to + 20 cmH_2_O, in 5 cmH_2_O increments. At each setting of the box pressure, the EB catheters were progressively inflated by 0.5 mL steps, from 0 mL until the minimum volume generating a balloon pressure of 30 cmH_2_O. Accurate TEP was defined as a value of 0 ± 1 cm H_2_O and accuracy rates were computed. Thus, within this range of accuracy, we defined the minimal (V_accuracy-min_) and maximal (V_accuracy-max_) volumes of accuracy, as previously reported^13^.The working volume of accuracy (V_working_) was defined as the difference between these two volumes. Of note, V_working_ was only computed during tests in which at least 2 balloon inflation volumes generated TEP within the range of accuracy.

### Transesophageal pressure measurements accuracy post positive pressure occlusion testing

We initiated experiments by setting box pressure to 0 cmH_2_O and inserting the esophageal catheter through the cable glands to position the balloon midway within the esophagus. Per each esophageal catheter, we inflated the balloon to the average V_accuracy-min_, as per the results of aforementioned tests. Following this, we conducted a procedure similar to the positive pressure occlusion test, applied in clinical settings^1^. This involved subjecting the chamber to four pressure increments up to 10 cm H_2_O, while simultaneously monitoring the pressure within the EB (Fig. E2). We calculated the ratio of the change in EB pressure to the change in chamber pressure (∆P_es_/∆P_box_). Our goal was to achieve a ∆P_es_/∆P_box_ ratio falling within the range of 0.8–1.2^1^. To accomplish this, we finely adjusted the EB inflation volume in increments/decrements of 0.1 mL. Subsequently, employing the determined EB volume, we varied the chamber pressure from − 20 to + 20 cmH_2_O, in increments/decrements of 5 cmH_2_O, and computed the TEP. Each test involved randomized increments or decrements in chamber pressure. Test were randomly conducted in triplicate per each type of catheter.

**Figure 2:**
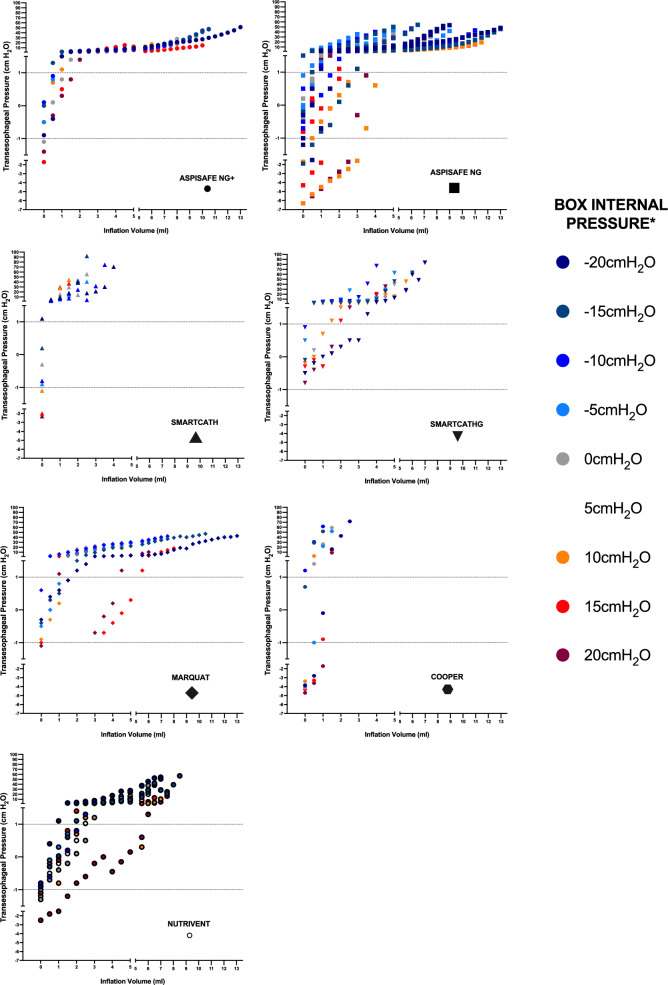
Transesophageal pressure, as defined as the difference between the esophageal balloon internal pressure and the box internal pressure, among different balloon inflation volumes and various box internal pressures. Per each graph, values are reported by different shapes, as reported under the name of each esophageal balloon catheter. The green bar between − 1 and 1 cmH_2_O highlights defined level of accuracy of transesophageal pressure. Median transesophageal pressure varied among catheter types (N = 2032, *p* < 0.001). Post hoc analyses: *p* < 0.05 SmartcathG versus Aspisafe NG+, Aspisafe NG, Marquat; *p* < 0.05 Nutrivent versus Smartcath, Aspisafe NG+, Aspisafe NG, Marquat. * The internal pressure color legend only reports the shape of Aspisafe NG+, please refer to other shapes for different catheters.

### Esophageal balloon outer diameter and length

We measured the outer diameter and length of each balloon using three randomly selected catheters, resulting in a total of 21 measurements (Table 1). After inflating the EB to its maximum insufflation volume according to the manufacturer's technical specifications, the length of the balloon was measured using the digital Vernier caliper. The outer diameter was measured by placing the caliper across the middle section of the balloon. As for the novel catheters manufactured by Aspisafe™, the balloon was inflated to an internal pressure of 40 cmH_2_O. This pressure was selected to enable complete balloon inflation, while minimizing the potential for rupture.

### Esophageal balloon elastance

Each EB has an external line to transmit the pressure to the proximal end. To measure the pressure, we connected the external line to a piezoresistive pressure transducer (PX181B–015C5V, Omega Engineering, Norwalk, CT, USA) and a 20-mL syringe using a 3-way stopcock. By using the syringe, the balloon was then progressively inflated in increments of 0.5 mL until reaching the minimum volume generating an internal pressure of 40 cmH_2_O. The number of steps required per each catheter varied based on the specific elastance of the balloon. Three samples per each type of catheter were tested. During the tests, we collected the following measurements: (1) Residual volume (V_res_), defined as the inflation volume before a linear increase in internal pressure occurs; (2) Balloon elastance, computed as the change in internal esophageal pressure divided by volume, during the linear increase in internal pressure; (3) Volume at which the internal pressure reached or exceeded 40 cmH_2_O (V_40cmH2O_)^13^.

### Statistical analysis

Continuous variables were described as median (interquartile range) and mean ± standard deviation, minimal and maximal values were also reported in comprehensive descriptive statistics tables. Categorical variables, such as TEP measurements within the range of accuracy were described as percentages and analyzed through Fisher exact test. Continuous variables, such as outer diameter, length, elastance, and TEP were evaluated using Kruskal–Wallis test to assess between-catheter differences. For each continuous variable, evidence of between-group differences was initially assessed by the global Kruskal–Wallis H test. Post-hoc analysis of statistically significant results (*p* ≤ 0.05) examined pairwise differences between catheter types. A Bonferroni correction was applied to hypothesis testing of pairwise comparisons to maintain a family-wise error rate of 0.05.

### Ethical approval

The study protocol underwent review by the Queensland University of Technology Ethics Committee, under application number 2020-3702-4115. It was determined that ethics approval could be waived, due to the utilization of esophagi sourced from commercially raised pigs at a slaughterhouse.

## Results

The manufacturing characteristics of each catheter are listed in Table E1. The Aspisafe NG, NG+ and the Nutrivent catheters were equipped with esophageal and gastric balloons. The Aspisafe NG+ esophageal and gastric balloons have been also designed to be sequentially inflated to reduce gastro-esophageal reflux. Among the seven catheters tested, four comprised an internal line for feeding and stomach aspiration. Overall, we tested 105 brand-new catheters and only one Nutrivent EB showed manufacturing abnormalities upon inflation (Fig. E3). None of the EBs presented manufacturing tears or observed air leakage upon inflation.

## Primary objective

### Appraisal of esophageal balloon volume for accurate transesophageal pressure measurement

Esophagi were used for testing after a median duration of 19 days (12–26) of storage in the cold preservation media, without significant differences among catheter types (N = 21, *p* = 0.651). Similarly, during tests, median fluid temperature of the pressurized box was 36.9 °C (36.5–37.5), with no significant difference among catheter types (N = 21, *p* = 0.477). Figure 2 show TEP measurements among tested EBs and box pressures. A total of 189 experiments were conducted, consisting of 7 catheters tested at 9 different internal chamber pressures, each repeated three times. As shown in Table 2, the V_accuracy-min_ median values ranged from 0.00 to 0.50 mL (*p* = 0.130), whereas V_accuracy-max_ ranged from 0.50 to 2.25 mL and differed significantly among tested EBs (*p* = 0.002). In line with these findings, V_working_ varied between 0.50 and 1.75 mL and significantly differed among tested balloons (*p* < 0.001). When the EBs were filled with the recommended inflation volumes, as provided by the manufacturers, the accuracy of TEP measurements varied across different catheter types (N = 446, *p* < 0.001). Specifically, accurate measurements were obtained in 11.9% of the values for Smartcath, 40.3% for SmartcathG, 23.1% for Marquat, 6.4% for Cooper, and 26.9% for Nutrivent.

**Table 2:** Descriptive statistics esophageal balloon inflation volumes within accurate transesophageal pressures—between − 1 and 1 cmH_2_O.

Catheter	Manufacturing recommended inflation volume (mL)	V_accuracy-min_ Mean ± SD (mL) Median (Q1-Q3) (mL)	V_accuracy-max_ Mean ± SD (mL) Median (Q1-Q3) (mL)	V_working_ Mean ± SD (mL) Median (Q1-Q3) (mL)
Aspisafe NG+	N/A	0.31 ± 0.50 0.00 (0.00–0.50)	1.31 ± 0.74 1.50 (0.50–2.00)	1.00 ± 0.52 0.50 (1.00–1.50)
Aspisafe NG	N/A	0.45 ± 0.98 0.00 (0.00–0.50)	1.33 ± 1.18 0.50 (0.50–2.00)	0.88 ± 0.65 0.50 (0.50–1.50)
SmartCathG	0.5–2.5	0.21 ± 0.57 0.00 (0.00–0.00)	2.14 ± 0.98 2.25 (1.50–3.00)	1.92 ± 1.07 1.75 (1.00–3.00)
SmartCath	0.5–2.5	0.30 ± 0.44 0.00 (0.00–0.50)	0.90 ± 0.41 0.50 (0.50–1.00)	0.60 ± 0.22 0.50 (0.50–0.50)
Marquat	0.5–3.0	0.46 ± 0.90 0.00 (0.00–0.50)	1.82 ± 1.36 1.50 (1.00–2.00)	1.35 ± 0.81 1.25 (1.00–1.50)
Cooper	1.0–2.0	0.00 ± 0.00 0.00 (0.00–0.00)	0.66 ± 0.28 0.50 (0.50–1.00)	0.66 ± 0.28 0.50 (0.50–1.00)
Nutrivent	4.0	0.96 ± 1.34 0.50 (0.00–2.00)	2.55 ± 1.76 1.00 (1.75–4.50)	1.59 ± 1.01 1.50 (1.00–2.00)

The minimal (V_accuracy-min_) and maximal (V_accuracy-max_) volumes were defined as the minimal and the maximal inflation volumes that resulted in a transesophageal pressure of − 1 and + 1 cmH_2_O. We define the working volume of accuracy (V_working_) as the difference between these two volumes.

*SD* standard deviation, *Q1* first quartile, *Q3* third quartile.

### Transesophageal pressure measurements accuracy post positive pressure occlusion testing

**Figure 3:**
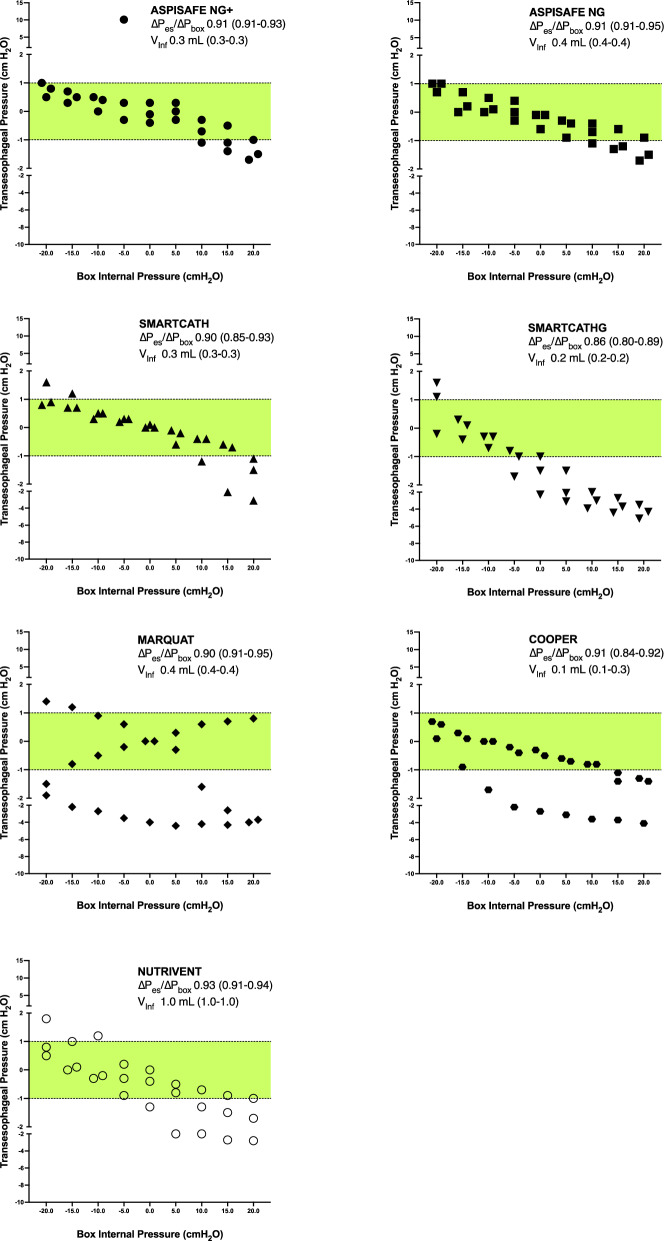
Transesophageal pressure, as defined as the difference between the esophageal balloon internal pressure and the box internal pressure, among different box internal pressures. The green bar between − 1 and 1 cmH_2_O highlights defined level of accuracy of transesophageal pressure. Median transesophageal pressure varied among catheter types (N = 189, *p* < 0.001). Post hoc analyses: *p* < 0.05 SmartcathG versus Aspisafe NG+, Aspisafe NG, Smartcath. Per each catheter type, median ∆P_es_/∆P_box_, ratio of the change in esophageal balloon pressure to the change in chamber pressure upon positive pressure occlusion test, V_inf,_ median volume of inflation of the esophageal catheter balloon (interquartile range) are showed on the top right of each graph. ∆P_es_/∆P_box_ was similar among catheters (N = 21, *p* = 0.113), while resulting inflation volumes significantly differed (N = 21, *p* < 0.001).

After a median storage duration of 15 days (11–16), we employed esophagi for our studies (N = 21, *p* = 0.995 among tested catheters). Median chamber temperature box was 37.0 °C (36.3–37.2 °C), (N = 21, *p* = 0.807 among catheter types). Upon positive pressure occlusion test, V_min_ was initially employed, and only increased in 3 and 1 out of 3 tests conducted with the Cooper and Nutrivent catheters, respectively (N = 21, *p* = 0.021 among tested catheters). Figure 3 depicts TEP among tested catheters and descriptive statistics of the ∆P_es_/∆P_box_ and applied inflation volumes. ∆P_es_/∆P_box_ was similar among catheters (N = 21, *p* = 0.113), while resulting inflation volumes significantly differed (N = 21, *p* < 0.001). The TEP accuracy rates for various catheter models are presented in Table 3. The Aspisafe NG exhibited the best accuracy rate of 81.4%, while the SmartcathG was accurate only in 37.0% of the measurements (N = 189, *p* < 0.001 among catheter types). When the box internal pressure was negative (-20 to -5 cmH_2_O), TEP measurements were accurate in 80.9% of the cases. Whereas, when the box internal pressure was positive (0 to 20 cmH_2_O), accurate pressure measurements were recorded in 47.6% of the cases. Considering all obtained measurements, the median TEP varied from − 1.5 cmH_2_O (IQR − 3.1 to − 0.3 cmH_2_O) of the SmartcathG, up to 0 cmH_2_O of the Aspisafe NG+ (IQR − 0.7 to 0.5 cmH_2_O) and Smartcath (IQR − 0.6 to 0.5 cmH_2_O) catheters (N = 189, *p* < 0.001 among catheters).

**Table 3:** Transesophageal pressure measurements accuracy post positive pressure occlusion testing.

Catheter	Total number of values within the range of accuracy/Total values (%)	Mean ± SD	Median (Q1-Q3)
Aspisafe NG+	21/27 (81.5)	0.19 ± 2.11	0.0 (− 0.7 to 0.5)
Aspisafe NG	22/27 (77.8)	− 0.27 ± 0.73	− 0.3 (0.2 to 0.9)
SmartCathG	10/27 (37.0)	− 1.71 ± 1.75	− 1.5 (− 0.3 to − 3.1)
SmartCath	20/27 (74.1)	− 0.14 ± 1.02	0.0 (− 0.6 to 0.5)
Marquat	20/27 (74.1)	− 1.33 ± 1.96	− 0.8 (− 3.5 to 0.6)
Cooper	16/27 (59.3)	− 1.10 ± 1.33	− 0.8 (− 1.7 to 0.0)
Nutrivent	17/27 (62.9)	− 0.58 ± 1.13	− 0.5 (− 1.3 to 0.1)

Accuracy rate and transesophageal pressure measurements significantly varied among tested catheters (N = 189, *p* < 0.001).

*SD* standard deviation, *Q1* first quartile, *Q3* third quartile.

## Secondary objectives

### Esophageal balloon outer diameter and length

Measurements of outer diameter and length of EB for each catheter type are shown in Fig. E4. Among different catheter types, the EB outer diameters (N = 21, *p* = 0.005) and lengths (N = 21, *p* = 0.004) varied significantly. In particular, Cooper and Smartcath balloons had the narrowest outer diameters, while the Aspisafe NG and NG+ balloons had the shortest lengths.

**Figure 4:**
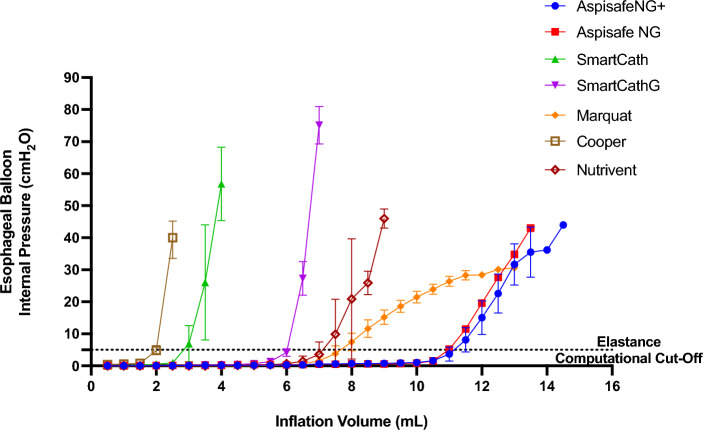
Esophageal balloon internal pressure of tested catheters per various inflation volumes. Descriptive statistics of the esophageal balloon elastance is reported in Table E3 and differed among catheter types (N = 87, *p* < 0.001).

### Esophageal balloon inflation volume and elastance

A total of 296 measurements were recorded. The median V_res_ varied broadly among catheters (N = 21, *p* = 0.006), as shown in Table E2. The Cooper had the lowest V_res_ of 0.5 mL (IQR 0.5–0.5), while the Aspisafe NG required 9.0 mL of V_res_ (IQR 6.5–10.0) to achieve an initial linear increase in internal pressure. Similarly, V_40cmH2O_ greatly varied among the catheter types (N = 21, *p* = 0.003). A statistically significant difference in balloons elastance was also found among catheter types (N = 87, *p* < 0.001) (Table E3 and Fig. 4). In particular, the Cooper balloon presented the highest median elastance of 15.7 cmH_2_O/mL (IQR 8.3–24.8), while the Aspisafe NG had the lowest elastance of 1.9 cmH_2_O/mL (IQR 1.1–2.6).

## Discussion

This study utilized a novel biomechanical 2.5-L model of the thoracic cavity, without cardiopulmonary organs to investigate several key aspects related to esophageal pressure monitoring and to investigate performance of the novel Aspisafe catheters in comparison with commercially available alternatives. The major findings of this study can be summarized as follows: Firstly, following the determination of inflation volumes from preliminary accuracy assessments—ranging from 0.1 to 1.0 mL—we found that 4 out of 7 tested catheters exhibited accuracy rates exceeding 75% in measuring TEP. However, when EBs were filled with volumes recommended by manufacturers, accuracy was only marginal. Secondly, the study unveiled significant disparities in the manufacturing characteristics of commercially available catheters utilized for esophageal pressure monitoring. This finding underscores a potential lack of standardized manufacturing practices within the industry. Lastly, the outer diameter, length, and material composition of the EB were identified as crucial factors affecting filling volumes and balloon elasticity. Notably, the novel Aspisafe catheters featured the largest polyurethane EBs, yet maintained elastance levels similar to previously developed models.

We have successfully developed a novel biomechanical model of the thoracic cavity. This innovative model intentionally excludes ventilatory tidal displacements and heartbeats, enabling more focused investigations into the accuracy of esophageal pressure monitoring catheters. Xiu-Mei et al. designed a bench model comprising increasing inner volumes (125–1000 mL) of glass chambers with different resulting chamber elastances^19^. While Yang et al. used a 5-L glass model of the pleural cavity^15^, comprising a 1-L rubber test lung. Conversely, Mojoli et al. used a 2-L plastic chamber without test lung^13^. Finally, Waltershapcher et al. appraised accuracy of various EBs, under static and dynamic conditions, into an air-tight chamber^14^. Our bench model distinguishes itself from previous designs, through notable advancements. A key improvement lies in the careful monitoring and control of chamber temperature and pressure. Additionally, we utilized swine esophagi that have undergone specialized processing to preserve their natural elasticity^20^ and to better mimic pressure transmission within the chest cavity. This approach enabled us to accurately simulate pressure transmission within the chest cavity, resulting in a model that closely mimics physiological conditions.

### Appraisal of esophageal balloon volume for accurate transesophageal pressure measurement

Numerous patient factors affect the accuracy of esophageal pressure monitoring as a surrogate for pleural pressure, including lung volumes, chest mechanics, body positioning, cardiac dimensions, and esophageal characteristics. Additionally, the inflation volume of the EB is critical, as improper inflation can lead to overestimation or underestimation of pleural pressure, impacting measurement accuracy^1^. Our research on the accuracy of TEP measurements across a wide range of negative and positive chamber pressures and balloon inflation volumes reinforces the findings of previous investigators. Furthermore, our study sheds light on the performance of newly developed catheters in this regard. Interestingly, our study supports the observations firstly made by Mojoli et al.^13^, demonstrating that positive and negative chamber/pleural pressures contribute to a higher likelihood of underfilled or overfilled balloons, respectively, thereby compromising the accuracy of the measurements. Indeed, as clearly shown in Fig. 2, low inflation volumes often underestimated positive box pressures, while high inflation volumes overestimated negative box pressures. Our study also revealed that inflating the balloons with volumes recommended by the manufacturers did not lead to a significant improvement in accuracy. Instead, our research suggests that adhering to lower inflation volumes yields superior results in terms of accuracy. Indeed, in comparison to previous findings^13,15,21^, both V_accuracy-min_ and V_accuracy-max_ values observed in our study were smaller. This discrepancy may be attributed to our inclusion of ex-vivo esophagi or the use of negative pressure chambers, whereas previous investigators had different setup and only focused on positive chamber pressures. Furthermore, we defined V_accuracy-min_ differently from the definition provided by Mojoli et al.^13^, who only included measurements above zero. In our study, we included scenarios where balloon inflation may not be necessary. This encompasses situations where balloon becomes overinflated, due to negative chamber pressure, or cases where narrow esophagi eliminate the requirement for balloon inflation.

### Transesophageal pressure measurements accuracy post positive pressure occlusion testing

Our results on TEP accuracy sheds light on the crucial role of EB inflation volume and design that can impact the accurate estimation of external pressure. These findings highlight the need for regular clinical validation of esophageal pressure monitoring. The positive pressure occlusion test^22^ has been widely proposed as a reliable validation method. Additionally, Mojoli et al. introduced an alternative approach utilizing the Nutrivent catheter in patients receiving positive pressure ventilation^23^. This method involves incrementally filling the balloon and selecting the volume that results in the largest esophageal pressure tidal swing. Ultimately, the difficulties associated with attaining precise TEP measurements underscore the significance of focusing on measurement trends over absolute values. To extrapolate the findings of our benchtop assessments to the clinical setting, we performed a dynamic pressure swing similar to the positive pressure occlusion test and observed that the novel Aspisafe balloons reliably transduced transesophageal pressure with an accuracy rate of up to 81.4% in our measurements. This performance aligned with existing commercially available alternatives, with the notable exception of the SmartcathG and Cooper balloons, which demonstrated comparatively lower performance levels. Importantly, as shown in Fig. 4, there was a clear trend toward underestimation of TEP values when the box pressure increased. This might have been related to the use of specific inflation volumes during our tests, which generally resulted in ∆P_es_/∆P_box_ values between 0.8 and 1.0.

### Clinical implications

Our findings carry important implications for clinicians and researchers involved in esophageal pressure measurements. It is crucial to recognize that following the manufacturer’s guidelines for balloon inflation may not guarantee optimal accuracy and could provide erroneous inferences for the ventilatory management of ventilated patients. Further clinical studies are needed to build upon these findings to investigate the underlying mechanisms and refine the recommended inflation volumes, thereby improving the overall accuracy and reliability of esophageal pressure measurements. We identified median V_accuracy-min_ and V_accuracy-max_ of the novel Aspisafe balloons in the range of 0.0 and 1.5 mL. Significantly, in the comparative analysis between the two catheter models, it was found that the Aspisafe NG+ design demonstrated a marginal enhancement in accuracy relative to the standard Aspisafe NG in acquiring TEP measurements. Notably, both catheters encompass EBs of similar dimensions and composition, differing only in the capability of the Aspisafe NG+ to be connected to a controller enabling sequential inflation of esophageal and gastric balloons to hinder gastro-esophageal aspiration and mitigate the risk of aspiration. However, the assessment of this feature’s effectiveness was not the primary focus of the manuscript and should be explored in future studies.

### Esophageal catheter balloon outer diameter, length and elastance

In our analysis, we found that EB length and diameter, as well as balloon volumes, were significantly different among the tested catheters, with some balloons being three times larger or longer than others. Moreover, there was an eight-fold disparity in the V_res_ required for some balloons compared to others, possibly creating hurdles in the conventional clinical applications for these catheters. In particular, the novel Aspisafe catheters presented the largest minimal inflation volumes. This can be attributed to the distinct purposes served by this novel catheter. Specifically, the esophageal and gastric balloons are intended to be inflated sequentially to prevent gastro-esophageal reflux, as opposed to solely measuring esophageal pressure. We also found an extensive range of elastance values among catheters, likely related to the balloon material and thickness. Most of the examined balloons were found to be constructed using polyethylene, a thermoplastic material well-known for its exceptional resistance and toughness. This observation is supported by the median elastance recorded for the polyethylene Cooper balloon, which reached a substantial value of 15.7 cmH_2_O/mL. However, it’s worth noting that the Nutrivent balloon, made of polyethylene, exhibited a lower elastance of 2.9 cmH_2_O/mL, likely attributed to the utilization of a thinner balloon material. In contrast, the Marquat balloon, composed of latex, a material known for its inherent elasticity, displayed a relatively lower median elastance of 2.2 cmH_2_O/mL. Whereas the novel Aspisafe balloons, manufactured from polyurethane, a material characterized by its notable elasticity, flexibility, and abrasion resistance, exhibited a median elastance between 1.9 and 2.1 cmH_2_O/mL. These findings underscore the potential advantages of polyurethane in providing desirable elastance properties for such balloons.

### Strengths and limitations

To the best of our knowledge, this is the first bench test of the novel Aspisafe EBs. In addition, our comprehensive analyses encompassed a large range of chamber pressures both negative and positive, which was often overlooked in previous studies. Nevertheless, some limitations should be emphasized. First, our setup was designed to accurately measure balloon inflation pressure, as well as the reaction of the surrounding esophageal tissue to pressure. This experimental setup allows for the comparison of outer and internal esophageal pressures to be studied, considering both the elastic properties of the balloon and the elastance of the esophageal wall. However, it is worth nothing that Orvar and colleagues^24^ clearly defined the pressure generated in-vivo by the esophageal wall when it is progressively distended by an EB. Since our experiment was conducted on ex-vivo porcine esophagi, we did not account for the potential active response of the esophageal wall’s smooth muscle that occurs in-vivo. A second major limitation of the model was that we did not assess the influence of ventilation and cardiac cycles on the pressure recordings. These factors should be considered when inferring our results into the clinical scenario. Thirdly, during TEP measurements, we utilized a 3-mL syringe to inflate the EB in increments of 0.5 mL, facilitated by connection to a 3-way stopcock. Consequently, disconnecting the syringe from the stopcock while inflating larger balloons may have resulted in some air leakage. Finally, although the Aspisafe NG+ is a catheter that also comprises a gastric balloon and the capability of sequential inflation of the esophageal-gastric balloons, we focused our investigation only on pressure measurement accuracy. Further in-vivo investigations are needed to evaluate additional features of this novel catheter.

### Conclusions

Comprehensive tests conducted in our novel biomechanical model, utilizing ex-vivo esophagi, showed that the novel Aspisafe balloons presented good level of accuracy in TEP measurements, consistent with several other commercially available alternatives. Accuracy was significantly impacted by the careful selection of inflation volumes and further validation through methods derived by the clinical positive pressure occlusion test. Finally, our findings highlight important heterogeneity in manufacturing characteristics of EBs, which could lead to enhanced or insufficient reliability in esophageal pressure monitoring during ventilatory support.

### Supplementary Information

Supplementary Information.

## Data Availability

The datasets used and/or analyzed during the current study are available from the corresponding author upon reasonable request.

## Abbreviations

ARDS: Acute respiratory distress syndrome
∆P_es_/∆P_box_: Ratio of the change in esophageal balloon pressure to the change in chamber pressure
EB: Esophageal balloon
IQR: Inter-quartile range
N: Number of analysed parameters
Q1: First quartile
Q3: Third quartile
SD: Standard deviation
TEP: Transesophageal pressure
V_accuracy-max_: Esophageal catheter balloon maximal volume of accuracy
V_accuracy-min_: Esophageal catheter balloon minimal volume of accuracy
V_inf_: Esophageal catheter balloon inflation volume
V_max_: Maximal inflation volume
V_res_: Residual volume
V_working_: Working volume
