# Supplementary material for: Comparative analysis of novel esophageal pressure monitoring catheters versus commercially available alternatives in a biomechanical model of the thoracic cavity
Source: Sci Rep. 2024 Apr 29;14:9771. doi: 10.1038/s41598-024-59790-1 (PMC11059186; doi:10.1038/s41598-024-59790-1)
Supplement: Supplementary file 1 — Supplementary Information. [file 41598_2024_59790_MOESM1_ESM.docx]

**ONLINE DATA SUPPLEMENT**

**Comparative Analysis of Novel Esophageal Pressure Monitoring Catheters vs. Commercially Available Alternatives in a Biomechanical Model of the Thoracic Cavity**

Gabriella Abbate, RN*^1,2^; Sebastiano Maria Colombo, MD^1,2^*; Clayton Semenzin, PhD^3^; Noriko Sato, RN^1^; Keibun Liu, MD, PhD^1^, PhD; Carmen Ainola, RN, MPhil^1^; Gabriele Fior^1,2,4^, MD; Angelo Milani, MD^1,5^; Nchafatso Obonyo, MD, PhD^1,6^; Nicole White, PhD^7^; Davide Chiumello, MD^8^; Jo Pauls, PhD; MD^9^; Jacky Y Suen, PhD^1,4,10^; John F Fraser, MB ChB, PhD, FRCP (Glas), FFARCSI, FRCA, FCICM, FELSO^1,4,11^ and Gianluigi Li Bassi, MD, PhD^1,4,11-13^

*GA and SMC equally contributed to this work

**Affiliations**

1. Critical Care Research Group, The Prince Charles Hospital, Brisbane, Australia
2. Fondazione IRCCS Ca' Granda Ospedale Maggiore Policlinico, Department of Anesthesia Critical Care and Emergency, Milan, IT
3. School of Engineering and Built Environment, Griffith University, Southport, Australia
4. University of Queensland, Brisbane, Australia
5. Department of Biomedical Sciences, Humanitas University, Pieve Emanuele (Milan), Italy.
6. Initiative to Develop African Research Leaders/KEMRI-Wellcome Trust Research Programme, Kilifi, Kenya
7. Australian Centre for Health Services Innovation and Centre for Healthcare Transformation, School of Public Health and Social Work, Queensland University of Technology, Brisbane, QLD, Australia.
8. San Paolo Hospital, Milan, Italy
9. School of Engineering and Built Environment, Griffith University, Brisbane, QLD, Australia
10. School of Pharmacy and Medical Sciences, Griffith University, Southport, Australia
11. Intensive Care Unit, St Andrew’s War Memorial Hospital, Spring Hill, QLD, Australia
12. Intensive Care Unit, The Wesley Hospital, Auchenflower, QLD, Australia
13. Wesley Research Institute, Auchenflower, QLD, Australia

***Table E1 Catheters manufacturing characteristics***

| Catheter | Catheter  diameter (Fr) | Catheter  length (cm) | Esophageal/Gastric Balloons material | Gastric feeding capability | Gastric balloon |
| --- | --- | --- | --- | --- | --- |
| AspisafeNG+ | 17 | 117 | Polyurethane | Yes | Yes |
| Aspisafe NG | 17 | 118 | Polyurethane | Yes | Yes |
| SmartCathG | 8 | 101 | Polyethylene | Yes | No |
| SmartCath | 16 | 114 | Polyethylene | No | No |
| Marquat | 6 | 78 | Latex | No | No |
| Cooper | 5 | 85 | Polyethylene | No | No |
| Nutrivent | 14 | 91 | Polyethylene | Yes | Yes |

**Table E1 Caption.** N/A, not available

***Table E2: Descriptive statistics of residual and maximal inflation volumes obtained by esophageal balloon elastance study***

| Catheter | V_res_ (mL)  Mean±SD  Median (Q1-Q3) | V_40cmH2O_ (mL)  Mean±SD  Median (Q1-Q3) |
| --- | --- | --- |
| Aspisafe NG® | 8.5±1.8  9.0 (6.5-10) | 13.5±0.0  13.5 (13.5-13.5) |
| AspisafeNG+® | 8.3±1.7  8.5 (6.5-10) | 13.6±0.8  13.5 (13.0-14.5) |
| SmartCath® | 1.5±0.1  1.5 (1.5-1.5) | 3.8±0.3  4.0 (3.5-4.0) |
| SmartCathG® | 4.2±1.9  5 (2.0-5.5) | 7.0±0.0  7.0 (7.0-7.0) |
| Marquat® | 6.0±0.9  6.5 (5.0-6.5) | 12.3±0.8  12.5 (11.5-13.0) |
| Cooper® | 0.5±0.1  0.5 (0.5-0.5) | 2.6±0.3  2.5 (2.5-3.0) |
| Nutrivent® | 5.2±0.3  5.0 (5.0-5.5) | 8.6±0.6  9.0 (8.0-9.0) |

**Table E2 Caption.** Data represent minimal (V_res_) and maximal (V_max_) inflation volumes at atmospheric pressure, as defined by the minimal volume prior to a linear increase in the pressure-volume curve, and the volume at which the internal balloon pressure ≥ 40 cmH_2_O. SD, standard deviation; Q3, third quartile; Q1, first quartile.

***Table E3 Descriptive statistics esophageal balloon elastance***

| Catheter | Number | Mean | SD | Max | Q3 | Median | Q1 | Minimum |
| --- | --- | --- | --- | --- | --- | --- | --- | --- |
| AspisafeNG+ | 16 | 1.87 | 0.93 | 3.20 | 2.60 | 1.95 | 1.08 | 0.49 |
| Aspisafe NG* | 17 | 1.95 | 0.91 | 3.18 | 2.67 | 2.08 | 1.16 | 0.49 |
| SmartCathG | 7 | 6.55 | 4.22 | 12.13 | 10.82 | 5.10 | 2.59 | 1.07 |
| SmartCath | 5 | 11.13 | 6.31 | 17.61 | 17.05 | 11.33 | 6.07 | 3.60 |
| Marquat^ | 29 | 2.01 | 0.52 | 2.71 | 2.35 | 2.17 | 1.69 | 0.83 |
| Cooper | 4 | 16.54 | 11.76 | 31.51 | 24.79 | 15.75 | 8.28 | 3.15 |
| Nutrivent | 9 | 3.14 | 1.72 | 5.34 | 4.87 | 2.99 | 1.45 | 1.06 |

**Table E3 Caption.** Balloon elastance was computed as the change in internal esophageal pressure/inflation volume, during the linear increase in internal pressure between the residual and maximal inflation volumes. Balloon elastance values are reported in cmH_2_O/mL. SD, standard deviation; Q3, third quartile; Q1, first quartile. * p<0.05 post hoc analyses vs SmartCath®; ^ p<0.05 post hoc analyses vs SmartCath® and Cooper®.

**Figure Legends**

**Figure E1:** The figure depicts tested esophageal catheters, from top to bottom: A) Aspisafe NG+® (Aspisafe, NY, USA);B) Aspisafe NG® (Aspisafe, NY, USA); C) SmartCath® (CareFusion Co., CA, USA); D) SmartCath-G® (CareFusion Co., CA, USA); E) Marquat® (Marquat Genie Biomedical, Boissy-Saint-Léger Cedex, France); F) Cooper® (Cooper Surgical, CT, USA); G) Nutrivent® (NutriVent, Sidam, Modena, Italy).

**Figure E2:** The figure depicts the third replication of the procedure employing the Nutrivent catheter balloon to simulate the positive pressure occlusion test applied in clinical settings (Akoumianaki, E. *et al.* The application of esophageal pressure measurement in patients with respiratory failure. *Am J Respir Crit Care Med* 189, 520–531 (2014)). In this instance, the Nutrivent was inflated with 1.0 mL of air. The chamber underwent four pressure increments up to 10 cm H_2_O (pressure target), while simultaneously monitoring the pressure within the esophageal balloon. Once the chamber pressure stabilized at the designated target level, as highlighted by the vertical lines depicting baseline and target pressure times of assessment, we calculated the ratio of the change in esophageal balloon pressure to the change in chamber pressure (∆P_es_/∆P_box_) and we computed a ∆P_es_/∆P_box_ ratio. Vertical lines represent the time points at which measurements were taken to calculate the ratios. This specific assessment resulted in ∆P_es_/∆P_box_ of 0.92.

**Figure E3:** Bulging of distal section of the balloon (arrow) upon inflation of the Nutrivent® esophageal catheter.

**Figure E4:** (A) Outer diameter and (B) length of esophageal balloons for each catheter type. The esophageal balloons outer diameters differ among catheters (N.21, p=0.005); similarly, balloon lengths were significantly dissimilar among catheters (N.21, p=0.004).

**Figure E1**

**
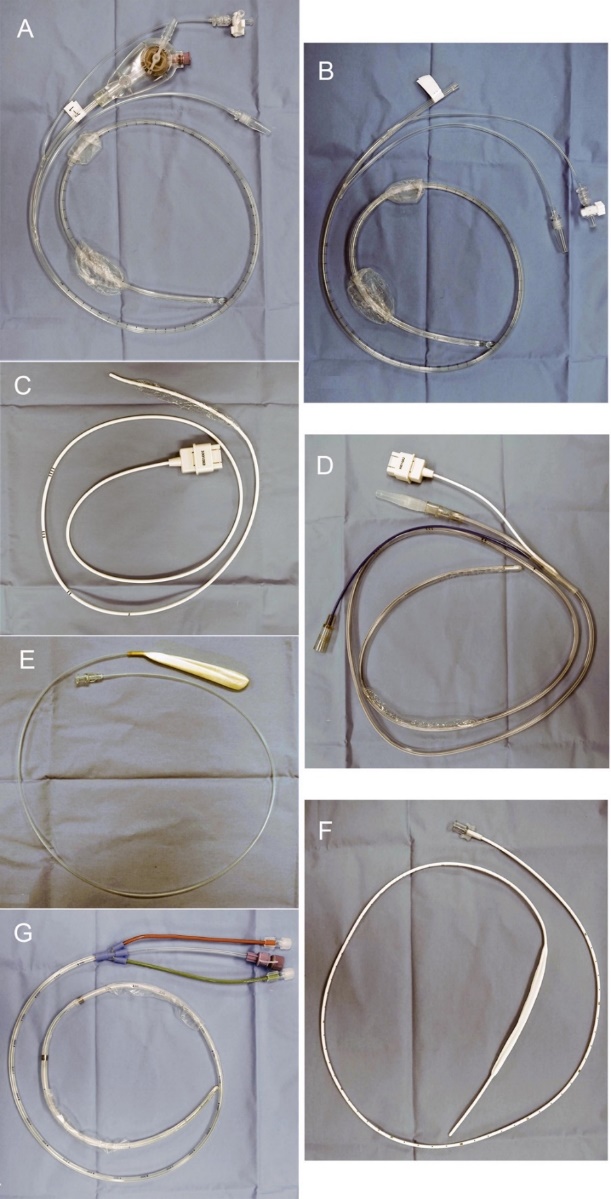
**

**Figure E2**

**Figure E3**

**
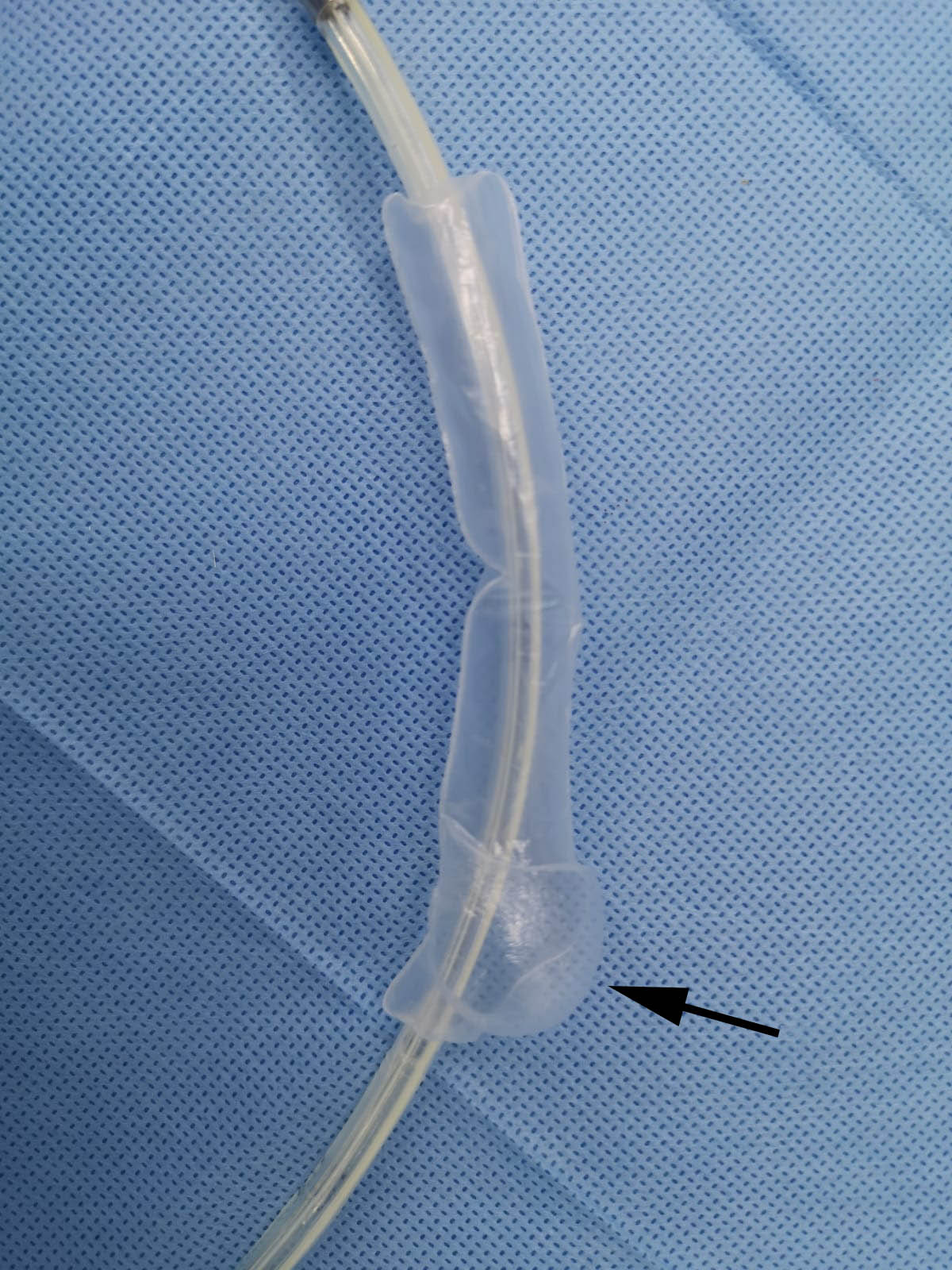
**

**Figure E4**
